# Frailty effects on non-demented cognitive trajectories are moderated by sex and Alzheimer’s genetic risk

**DOI:** 10.1186/s13195-019-0509-9

**Published:** 2019-06-21

**Authors:** Sherilyn Thibeau, Kirstie McDermott, G. Peggy McFall, Kenneth Rockwood, Roger A. Dixon

**Affiliations:** 1 Department of Psychology, University of Alberta, P217 Biological Sciences Building, Edmonton, AB Canada; 2 Neuroscience and Mental Health Institute, University of Alberta, 4-120 Katz Group Centre, Edmonton, AB Canada; 3 Department of Geriatric Medicine, QEII Health Sciences Centre, Dalhousie University, VG Site, Suite 442 Bethune Building, 1276 South Park Street, Halifax, NS Canada

**Keywords:** Frailty, Executive function, Episodic memory, Speed, Sex, *Apolipoprotein E*, Victoria Longitudinal Study

## Abstract

**Background:**

Age-related frailty reflects cumulative multisystem physiological and health decline. Frailty increases the risk of adverse brain and cognitive outcomes, including differential decline and dementia. In a longitudinal sample of non-demented older adults, we examine whether (a) the level and/or change in frailty predicts trajectories across three cognitive domains (memory, speed, and executive function (EF)) and (b) prediction patterns are modified by sex or Alzheimer’s genetic risk (*Apolipoprotein E* (*APOE*)).

**Methods:**

Participants (*n* = 632; *M* age = 70.7, range 53–95; 3 waves) were from the Victoria Longitudinal Study. After computing a frailty index, we used latent growth modeling and path analysis to test the frailty effects on level and change in three latent variables of cognition. We tested two potential moderators by stratifying by sex and *APOE* risk (ε4+, ε4-).

**Results:**

First, frailty levels predicted speed and EF performance (level) and differential memory change slopes. Second, change in frailty predicted the rate of decline for both speed and EF. Third, sex moderation analyses showed that females were selectively sensitive to (a) frailty effects on memory change and (b) frailty change effects on speed change. In contrast, the frailty effects on EF change were stronger in males. Fourth, genetic moderation analyses showed that *APOE* risk (e4+) carriers were selectively sensitive to frailty effects on memory change.

**Conclusion:**

In non-demented older adults, increasing frailty is strongly associated with the differential decline in cognitive trajectories. For example, higher (worse) frailty was associated with more rapid memory decline than was lower (better) frailty. These effects, however, are moderated by both genetic risk and sex.

**Electronic supplementary material:**

The online version of this article (10.1186/s13195-019-0509-9) contains supplementary material, which is available to authorized users.

## Background

Age-related frailty reflects cumulative multisystem physiological and health decline [1]. Frailty increases the risk of adverse brain and cognitive outcomes, including differential decline and dementia [2, 3]. Recently, understanding frailty and its impact has become a priority in clinical and research settings [4]. Currently, there are two main methods of physical frailty measurement: (a) a phenotype model and (b) an accumulation of deficits (frailty index) model. The phenotype model defines frailty as the presence of three of five criteria: unintentional weight loss, exhaustion, weakness, slow gait, and low physical activity [5]. A frailty index is formed by the ratio of health deficits present in an individual to the total number of potential deficits measured [6]. In the current research, we use the frailty index (FI) for four main reasons: (a) the FI shows greater predictive value than other frailty measures for populations in community settings [7], (b) it is considered one of the most robust frailty assessment tools [8], (c) it is unidimensional and has high constructive validity [9], and (d) the total FI score (more than individual health deficits) has been found to be more predictive of adverse outcomes [10].

Cognitive variables are often included in frailty indices. However, elevated frailty and cognitive impairment are distinguishable facets of aging that interact in the cycle of age-related decline. Accordingly, increasing frailty may be an indicator of future cognitive decline and impairment [11]. In fact, Armstrong and colleagues [12] examined the association between a frailty index and global cognition. Their results indicated that higher (worse) frailty was associated with worse cognition at baseline and a faster rate of cognitive decline [12]. Notably, longitudinal relationships between frailty indices and specific cognitive domains are few. One study by Rolfson and colleagues [13] indicated that over a 3-year period, worse frailty index scores were associated with reduced neurocognitive speed performance. We expand and contribute to this area of research by examining the longitudinal relationships between a frailty index and three distinct cognitive domains, namely, memory, speed, and executive function (EF). However, as both cognitive aging and the accumulation of health deficits involve many complex, heterogeneous, interacting factors and processes [4, 14], the relationship between frailty and cognitive decline may be further influenced by other risk factors for Alzheimer’s disease (AD), such as sex or Apolipoprotein E (*APOE*) genetic risk.

*APOE* has three major isoforms (ɛ2, ɛ3, and ɛ4), with the ɛ4 increasing the risk of cognitive decline and AD in a dose-dependent fashion [15]. The isoforms differentially regulate systems involved in AD pathology including (a) amyloid beta aggregation and clearance, (b) neuroinflammation, (c) lipid transport, and (d) glucose metabolism (Bennet et al. 2007, [15]). On its own, the ɛ4 allele is an established risk factor for cognitive decline in normal aging, mild cognitive impairment, and AD [15, 16]. Additionally, the ɛ4 allele has been considered a “frailty allele” and has been included as an indicator of frailty in some frailty indexes [17]. An independent association between *APOE* and frailty has not been demonstrated [18]; therefore, *APOE* may operate by way of interaction or moderation when considered in relation to cognition. In fact, *APOE* has been found to exert moderating effects on the relationship between single health factors (i.e., vascular health and pulse pressure) and cognitive change with aging [19, 20]. As such, it is possible that *APOE* may moderate the effect of frailty on cognitive performance and change.

Sex differences in frailty have garnered a great deal of attention in the literature. Results of a recent meta-analysis showed that females had higher frailty index scores than males at all ages but a lower mortality rate at any given level of frailty or age, indicating that frailty is more lethal in males than females [21]. Additionally, sex differences in level and change trajectories are evident across many cognitive domains, with females demonstrating generally higher levels of performance and greater resilience to age-related cognitive decline than men [22]. Notably, females are disproportionately affected by AD in severity, progression, and prevalence [23], and female carriers of the *APOE* ɛ4 risk allele are at a higher risk for AD than male carriers [24]. Moreover, females with *APOE* ɛ4 and higher levels of beta-amyloid burden experienced faster rates of cognitive decline than their male counterparts [25]. Taken together, sex may also influence the relationship between age-related cognitive performance and decline and frailty.

### Research goals

The overall purpose of this study was to examine the relationships of both level (at a statistical centering age) and slope (longitudinal change) between frailty and three cognitive domains: (a) episodic memory, (b) neurocognitive speed, and (c) executive function (EF) as moderated by two non-modifiable risk factors for AD (i.e., sex and *APOE* genetic risk). We assembled a 3-wave dataset, covering a 40-year age span (53–95) and used structural equation modeling to investigate three research goals (RGs). For RG1, we examined how frailty (level or change) affected the level and change in the three latent cognitive variables. For RG2, we examined whether *APOE* (risk, non-risk) moderated the level and longitudinal frailty-cognition relationships. For RG3, we examined whether sex moderated the level and longitudinal frailty-cognition relationships.

## Methods

### Participants

Participants were community-dwelling older adult volunteers of the Victoria Longitudinal Study (VLS; see Table 1 for demographic information). The VLS is a Canadian, large-scale, long-term investigation of neurocognitive aging, impairment, and dementia as influenced by genetic, biomedical, biological, health, lifestyle, and other factors [26]. Three main sequential samples (initially aged 53–95 years) are followed at about 4-year intervals (*M* = 4.4-year interval). All participants provided written informed consent, and all data collection procedures were in full and certified compliance (annually) with the Health Research Ethics Board at the University of Alberta. As the focus of this study was to examine the change in cognition as moderated by a genetic variant, participants were limited to a source subsample who had provided biofluid for genotyping between 2009 and 2011 (*n* = 695). This source subsample consisted of current subsets of three equivalent sequential cohorts, with present data collection occurring in the 2001–2015 period. The VLS cohorts were from sample 1 (waves 6, 7, and 8), sample 2 (waves 4 and 5), and sample 3 (waves 1, 2, and 3). The total individualized duration is up to 9 years [27]. The wave-to-wave retention rates by sample ranged from 77 to 90% (see Table 2 for attrition rates). We note that those who did not return for a third wave of data collection (*n* = 44) had higher levels of frailty and lower cognitive performance at the second wave of measurement than returners. The following exclusionary criteria were applied at baseline to the source sample: (a) a diagnosis of AD or dementia (*n* = 0), (b) missing data at all three waves across any one of the 50 measures used to calculate frailty index (*n* = 40), and (c) missing data at all three waves across any one of the 4 measures used to calculate the memory, speed, or EF latent variable (*n* = 23). The final study sample was comprised of 632 adults at baseline (*M* age = 70.7, range = 53.25–95.45; 66.7% female; see Table 1 and Additional file 1: Figure S1 for the study diagram).

**Table 1:** Baseline descriptive statistics by *APOE* genotype

*APOE*	ɛ4+ (risk)	ɛ4- (non-risk)
n	146	456
Age	69.85 (8.34)	71.01 (8.86)
Range	55.0–87.0	53.0–95.0
Sex (% female)	63.7	66.9
Education (years)	15.63 (3.02)	15.15 (3.00)
Range	8.00–24.00	5.0–23.00
MMSE	28.80 (1.19)	28.70 (1.24)
Range	25.00–30.00	34.00–30.00
Frailty Index Score	0.12 (0.07)	0.13 (0.07)
Range	0.01–0.32	0.01–.042

Note. Results presented as Mean (Standard Deviation). *MMSE* Mini Mental State Exam

**Table 2:** Attrition rates per sample and wave

		Wave 1	Wave 2		Wave 3	
			Return	Non Return	Return	Non Return
Sample One	*n*	58	49	9 (15.5%)	38	11 (22.4%)
Sample Two	*n*	179	146	33 (18.4%)	–	–
Sample Three	*n*	394	333	61 (15.4%)	300	33 (10%)

*Note*. Results presented as n (% attrition). Due to ongoing data collection, Sample two did not contribute a third data point to this study

### Measures

#### Frailty index

For each participant, the frailty index tallied the total number of physical health deficits from 50 variables (see Table 3) which previous work suggests is sufficient for accurately predicting adverse outcomes [28]. The items collected included self-report data, physical examination, and formal tests with standardized scales. All frailty items were consistent with those included in previous frailty indexes [29–33]. As cognitive performance and change were the primary outcomes, all cognitive-related measures or reports were excluded from the present frailty index.

**Table 3:** List of variables used to construct the 50-item frailty index

	Frailty measures	Coding
SR	Stroke	0 = no; 0.33 = yes, not serious; 0.67 = yes, moderately serious; 1 = yes, very serious
	Thyroid condition	
	Arthritis (rheumatoid and/or osteo-)	
	Osteoporosis	
	Cancer	
	Asthma	
	Migraines	
	Stomach ulcer	
	Kidney or bladder trouble	
	Gastrointestinal problems (colitis/diverticulitis, gall bladder trouble, and/or liver trouble)	
	Bronchitis or emphysema	
	Diabetes	
	High blood pressure	
	Sex-related health problems (i.e., gynecological problems or prostate problems)	
	Anemia	
	Drug and/or alcohol dependence	
	Spinal condition and/or back trouble	
	Hardening of arteries (i.e., atherosclerosis)	
	Heart trouble	
	Other conditions (up to three)	
SR	Number of medications	0 = 0–3; 0.5 = 4–7; 1 = 8+
SR	Subjective health relative to a perfect state of health	0 = very good; 0.25 = good; 0.50 = fair; 0.75 = poor; 1 = very poor
	Eyesight relative to age group	
	Hearing relative to age group	
SR	Health has affected ability to do chores	0 = no change, improved, N/A; 0.25 = slightly reduced; 0.50 = moderately reduced; 0.75 = drastically reduced; 1 = gave up doing activity
	Health has affected ability to get around town	
	Health has affected ability to do mental recreational activities	
	Health has affected ability to do physical recreational activities	
	Health has affected ability to do hobbies	
	Health has affected ability to socialize	
	Health has affected ability to travel	
SR	Stay at home but in chair most of the time	0 = no; 1 = yes
SR	Number of times sick in bed all day in the past year	0 = 0–3; 1 = 4+
SR	Number of timse confined to hospital in the past year	0 = 0; 0.5 = 1–2; 3+ = 1
SR	Feeling short of breath	0 = no; 1 = yes
SR	Use of a walker, cane, or wheelchair	0 = no; 1 = yes
M	Resting heart rate (bpm)	0 = 60–99; 1 = < 60 or 100+
M	Pulse pressure (mmHg)	0 = 32.13–63.90; 0.5 = 64–75.9; 1 = 76+
M	Peak expiratory flow (L/min)	Men: 0 = > 340, 1 = ≤ 340 Women: 0 = > 310, 1 = ≤ 310
M	Body mass index (kg/m^2^)	0 = 18.5–25; 0.5 = 25 to < 30; 1 = < 18.5 or ≥ 30
M	Grip strength (kg)	Men: for BMI ≤ 24, GS ≤ 29; for BMI 24.1–28, GS ≤ 30; for BMI > 28, GS ≤ 32 Women: for BMI ≤ 23, GS ≤ 17; for BMI 23.1–26, GS ≤ 17.3; for BMI 26.1–29, GS ≤ 18; for BMI > 29, GS ≤ 21
M	Timed walk	0 = ≤ 10 s; 1 = > 10 s
M	Timed turn	0 = < 90th percentile; 1 = within the 90th percentile
M	Finger dexterity	0 = < 90th percentile; 1 = within the 90th percentile
SR	CES-D “during the past week, my sleep was restless”	0 = rarely or none of the time; 0.33 = some or a little of the time; 0.67 = occasionally or a moderate amount of the time; 1 = most or all of the time
SR	CES-D “during the past week, I felt depressed”	
SR	CES-D “during the past week, I felt lonely”	
SR	CES-D “during the past week, I could not get going”	
SR	Bradburn negative affect (restless, lonely, bored, depressed, upset due to criticism)	0 = no to all; 0.2 = yes to one; 0.4 = yes to two; 0.6 = yes to three; 0.8 = yes to four; 1 = yes to all
SR	Physical activity at least 2–3 times per week	0 = yes; 1 = no

*SR* self-reported, *M* measured, *CES-D* Center for Epidemiological Studies Depression Scale

The frailty index was constructed by first recoding each variable to an interval between 0 and 1 (see Table 1). For variables with two possible responses, scores were either 0 (deficit absent) or 1 (deficit present). Variables with four or five possible responses (e.g., subjective health responses included “very poor,” “poor,” “fair,” “good,” and “very good”) had scores that reflected a range between 0 and 1 (e.g., 0.00, 0.25, 0.50, 0.75, 1.00). For all participants, we calculated the frailty index as *x*/50, where *x* was the individual participant’s number of deficits (i.e., an individual with no deficits would have a frailty score of 0). In this sample, the frailty index means ranged from 0.13 to 0.53 at each wave (see Table 1), which is similar to previous studies [34].

#### DNA extraction and genotyping

As described in previous studies [35], the VLS collects saliva according to the standard biofluid collection, stabilization, and preparation procedures from DNA Genotek technology. Genetic analyses included genotype categorization based on the presence or absence of the risk allele. *APOE* genotype was divided into dichotomous categories: ε4+ (risk) consisted of ε4/ε4 and ε3/ε4 allele combinations and ε4- (non-risk) consisted of ε2/ε2, ε2/ε3, and ε3/ε3 allele combinations. For all analyses including *APOE*, we removed the genotype which combines the risk and protective alleles (ɛ2/ɛ4; *n* = 30) [27]. The genotypic distribution for *APOE* was in Hardy-Weinberg equilibrium, *χ*^2^ = .89.

#### Measures for the cognitive latent variables

The memory, speed, and EF tests included in the current study have been frequently used and validated with older adults in the VLS (and other studies). Citations indicate sources for established measurement attributes, structural characteristics, and sensitivity to health and neurological factors in older adult populations. For each set of manifest indicators, we calculated a latent variable to represent the construct.

##### Episodic memory

We calculated a robust latent variable comprised of four manifest indicators from two memory tasks [20]: Word Recall score on list 1 and score on list 2 and Rey Auditory Verbal Learning Test list B1 and list A6.

##### Word recall

Two lists of 30 content diverse English words were used to test immediate recall in a rotated design. Participants were given 2 min to study each list and 5 min to write as many words as they could recall [36].

##### Rey auditory verbal learning test

A list of 15 nouns was read aloud and immediately recalled; this process was repeated for five trials (A1–A5). Then, a list (B1) of 15 unrelated nouns was read aloud and immediately recalled, measuring free recall. Then, the participant was asked to recall the first list of nouns (A6), measuring recall after interference [37].

##### Speed

We calculated a robust speed latent variable comprised of four manifest indicators from four speed tasks following established procedures [35]. The tasks were simple reaction time, choice reaction time, lexical decision, and sentence verification. Because each of the speed measures varied in complexity, we applied validated correction procedures with specific lower and upper limits as follows: (a) simple reaction time, 150 ms; (b) choice reaction time, 150 ms and 4000 ms; (c) lexical decision, 400 ms and 10,000 ms; and (d) sentence verification, 1000 ms and 20,000 ms. Subsequently, trials were removed if they fell 3 standard deviations above or below the mean.

##### Simple reaction time

Participants were presented with a warning stimulus (***) followed by a signal stimulus (+) in the middle of the computer screen and asked to press a key as quickly as possible when the signal stimulus appeared. Fifty trials were administered, and the latency of the 50 trials was used for analysis [38].

##### Choice reaction time

A grid of (+) was presented on the computer screen; after a 1000-ms delay, one of the (+) was changed to a square, and participants were asked to indicate the location of the square using a matching arrangement of keys on the response console. The dependent measure was the average latency across 20 trials [39].

##### Lexical decision

A string of five to seven letters was presented on the computer screen. Participants were asked to identify as quickly as possible whether the letters formed an English word. The average latency across 60 trials was used for analysis [39].

##### Sentence verification

A sentence was presented on the computer screen, and participants were asked to identify as quickly as possible the plausibility of the sentence. The average latency across 50 trials was used for analysis [39].

##### Executive function

We calculated a robust EF latent variable comprised of four manifest EF indicators [40]: Hayling Sentence Completion Test, Stroop Test, Brixton Spatial Anticipation Test, and Color Trails Test part two.

##### Hayling sentence completion

In section A, participants listened to 15 sentences read aloud with the last word missing, completing the sentence in a way that made sense and as quickly as possible. In section B, participants again listened to 15 sentences read aloud with the last word missing, completing the sentence quickly with a word that was unrelated or unconnected to the sentence. Response speed on both sections and errors within section B were used to create an overall scaled score (ranged from 1 [impaired] to 10 [very superior]) [41].

##### Stroop

In part A, participants named the color of 24 dots (blue, green, red, or yellow) as quickly as possible. In part B, participants named the ink color of 24 words (e.g., “when”). In part C, participants named the ink color of color names (blue, green, red, or yellow) by ignoring the printed word and instead stating the color of the ink (e.g., if the word blue was printed in red ink, the correct answer was red). Scores were calculated from the interference index ([part C time − part A time]/part A time) which reflects slowing in response to interference [42].

##### Brixton spatial anticipation Test

Participants deduced simple and changing patterns by predicting the movement of a blue dot among ten possible positions on a page, which followed patterns that came and went without warning. The total errors were recorded (maximum 54) and converted to scaled scores. An overall standardized scale resulted in scores ranging from 1 (impaired) to 10 (very superior) [41].

##### Color trails test part two

Participants connected the numbers 1 to 25 by alternating between pink and yellow circles while disregarding the numbers in circles of the alternate color. The latency score to complete the task was used for analysis (lower scores indicated better performance) [43].

### Statistical analyses

Analyses pertaining to our three RGs included confirmatory factor analyses, longitudinal measurement invariance, latent growth modeling, and moderation analyses through structural equation modeling (SEM) using Mplus 7 [44]. Consistent with recommended standards and other VLS research, chronological age was coded as a continuous variable and used as the metric of change for all analyses. Age was centered at age 75, the approximate mean of the 40-year span of data, and a commonly observed inflection period in non-demented cognitive aging [45, 46]. We used robust maximum likelihood estimation methods based on all available information from every variable included in the covariance matrix, to estimate any missing values [47].

#### Foundational analyses

We conducted several analyses to test and confirm the basic characteristics of the data. Analyses for each cognitive variable were conducted separately (i.e., confirmatory factor analysis, measurement invariance, and latent growth modeling analyses were conducted (in that order) for the EF latent variable, then for memory, and finally for speed). All model testing, model fit indices, and chi-square difference tests are reported in Additional file 1: Tables S1-S4.

First, confirmatory factor analysis was used to test whether four EF manifest variables fit a single-factor EF construct and whether this single-factor EF variable fit the data for these participants. Second, longitudinal measurement invariance was tested, which examines whether (a) the EF measures used represent the same latent construct at each time of measurement, (b) each EF latent variable measures the same construct, and (c) whether there are mean differences in the EF latent variable means at each time point (see Additional file 1 for further details). Third, latent growth modeling was used to test the variability in intra-individual patterns of change over time for frailty, and then separately for each cognitive domain (see Additional file 1 for model testing procedure, fit indices, and results). The best growth models established variability in both level and change over time for frailty and each cognitive domain (one growth model each for the following: frailty, memory, speed, and EF; for a total of four growth models) and were used in the analyses for RG1–RG3 (see Additional file 1: Tables S1-S4).

##### Analyses for RG1: Independent effect of frailty on, separately, memory, speed, and EF

Using the best growth models from the foundational analysis, we estimated three parallel process models to test whether the level or change in frailty predicted cognitive performance or change. We estimated three parallel process models to see whether (a) the level of frailty predicted either level or change in (separately) memory, speed, or EF, and (b) the change in frailty predicted change in (separately) memory, speed, or EF (see Additional file 1 for parallel process model description and testing procedures, and Additional file 1: Figure S1 for parallel process model diagram).

##### Analysis for RG2: Moderation of the frailty-cognition relationships by sex

A series of steps to test sex moderation was followed. First, a model which tested the effect of frailty (intercept) regressed on both level memory (intercept) and change in memory (slope), and frailty change (slope) regressed on memory change (slope) was estimated, with all the parameter estimates constrained to be equal across sex (i.e., female and male) groups. Second, the parameters were free to vary between sex groups to examine moderation. Evidence of moderation was indicated by a significant deviance test which compared the fully constrained model to the unconstrained model [47]. This indicated a model in which the effect of frailty on memory performance and change was different for males, and females fit the data better than a model for which the effect of frailty on memory level and change was the same for both groups. The same series of steps was used to test sex moderation of the frailty-speed, and then the frailty-EF relationships.

##### Analyses for RG3: Moderation of the frailty-cognition relationships by *APOE*

We used the *APOE* groups (i.e., risk and non-risk) and applied the aforementioned analytical moderation steps to examine *APOE* moderation of the frailty-memory relationships. The same series of steps was used to test *APOE* moderation again for the frailty-speed and then the frailty-EF relationships.

## Results

### Foundational analyses

**Fig. 1:**
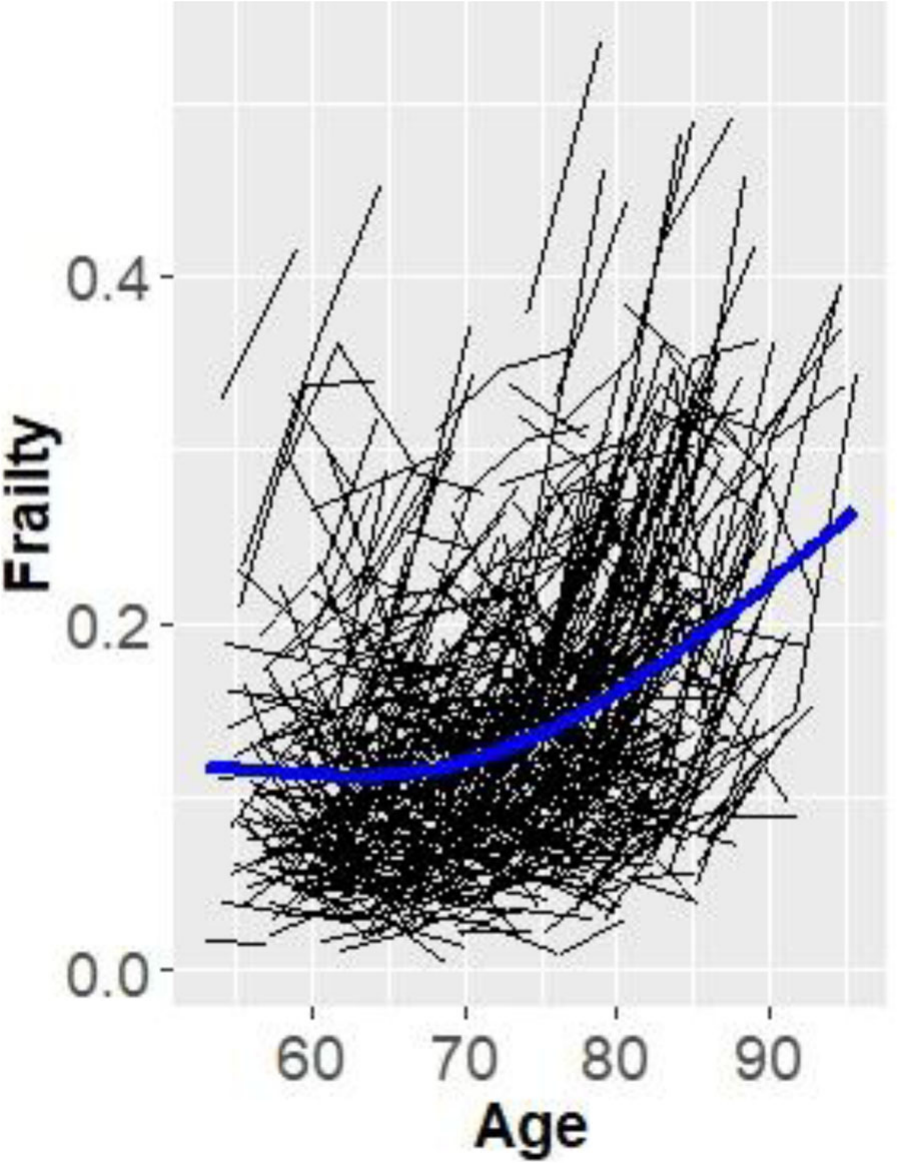
Individual frailty trajectories across a 40-year band of aging with a group mean trajectory line (final growth model random intercept, random slope; *D* = 37.2, Δ*df* = 2, *p* < .001)

In foundational analyses, we separately tested and verified longitudinal invariance for the one-factor memory, speed, and EF latent variables. The frailty, EF, speed, and memory growth models were computed over a 40-year period. The results of the confirmatory factor analysis and measurement invariance testing indicated that for this sample of participants, the best fitting models were unidimensional models with mean differences at the latent level for EF, memory, and speed. The latent growth modeling results indicated that for speed, memory, and EF, there was significant (a) variability in the level of cognitive performance, (b) decline in the cognitive scores over time, and (c) variability in change over time. The specific model fit indices, model comparisons, and distribution of cognitive trajectories are presented in Additional file 1: Table S1–S4; Figure S2–S5. Additionally, the latent growth model for frailty (higher score = worse) indicated that individuals varied in the level of frailty, exhibited a significant increase in frailty scores (*M* = 0.034, *p* < 0.01), and showed variable patterns of decline *(b* = 0.001, *p* < 0.01; see Fig. 1). As can be seen in Fig. 1, (a) the full distribution of frailty index trajectories reveals variability in level and slope and (b) the group mean trajectory curve (in bold) documents the gradual increase in frailty over the 40-year band of aging.

### RG1: Independent effect of frailty on cognition

#### Frailty predicting memory

**Fig. 2:**
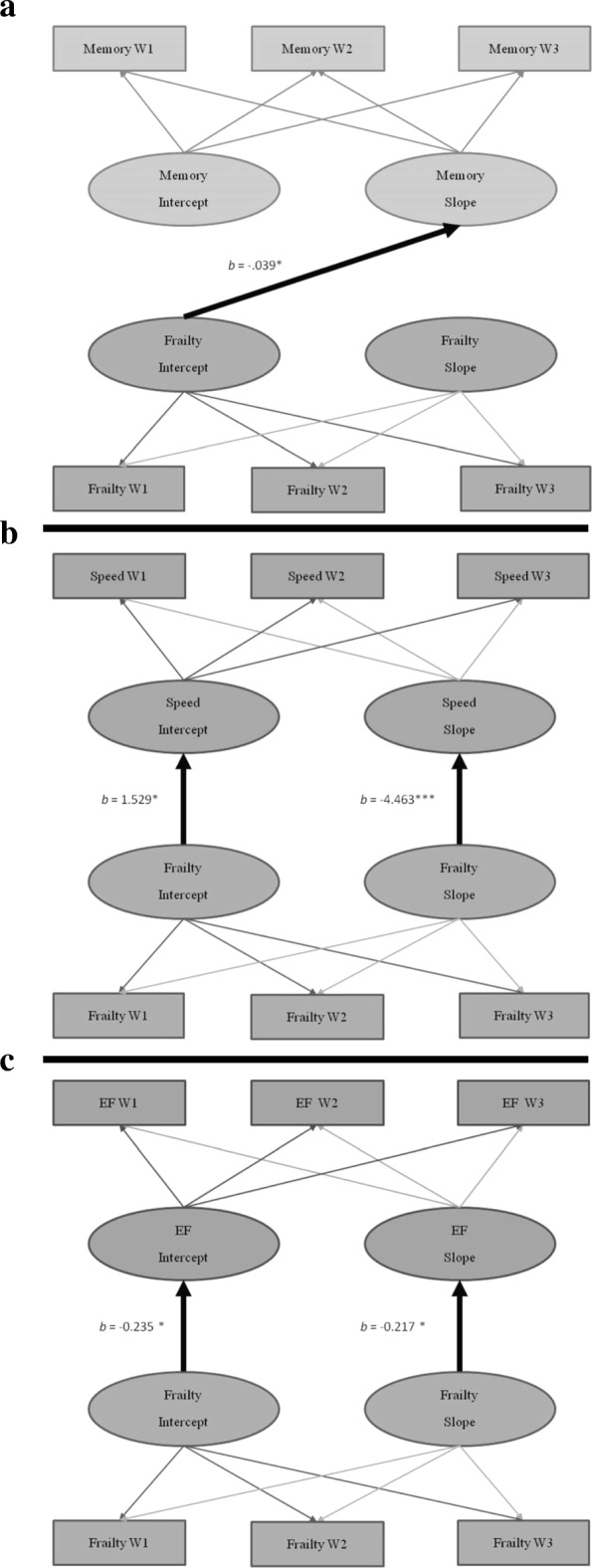
**a**–**c** Frailty-cognition parallel process models. Age in years was the metric of change. The age variable was centered at 75 years. *p < .05, ***p < .001. **a** is the Frailty-Memory parallel process model. **b** is the Frailty-Speed parallel process model. **c** is the Frailty-Executive Function (EF) parallel process model

Although baseline frailty did not predict the baseline level of memory performance (*b* = − 0.435, *p* = 0.189), it significantly predicted the rate of memory change (*b* = − 0.039, *p* = 0.032). Change in frailty did not predict the change in memory (*b* = − 0.032, *p* = 0.946). In sum, higher (worse) frailty was associated with more rapid memory decline than was lower (better) frailty (see Fig. 2a).

#### Frailty predicting speed

Frailty level significantly predicted the level of speed performance (*b* = − 1.529, *p* = 0.041) but did not predict the rate of change (*b* = − 0.03, *p* = 0.436). Notably, the change in frailty significantly predicted the change (slowing) in speed performance (*b* = − 4.463, *p* < 0.001). In sum, higher (worse) frailty was associated with slower levels of speed performance (see Fig. 2b). Additionally, a more rapid increase in frailty was associated with a more rapid decrease in speed.

#### Frailty predicting EF

Frailty level significantly predicted the level of EF performance (*b* = − 0.235, *p* = 0.019) but did not predict the rate of EF change (*b* = − 0.01, *p* = 0.151). In addition, the change in frailty significantly predicted the change in EF performance (*b* = − 0.217, *p* = 0.049). In sum, higher (worse) frailty was associated with lower levels of EF performance than was lower (better) frailty (see Fig. 2c). Additionally, a more rapid increase in frailty was associated with a more rapid decrease in EF.

### RG2: Moderation of the frailty-cognition relationships by sex

We conducted six sets of moderation analyses to examine whether sex differentially moderated the previously observed frailty-memory, frailty-speed, and frailty-EF relationships.

#### Sex moderation of the frailty-memory relationship

**Fig. 3:**
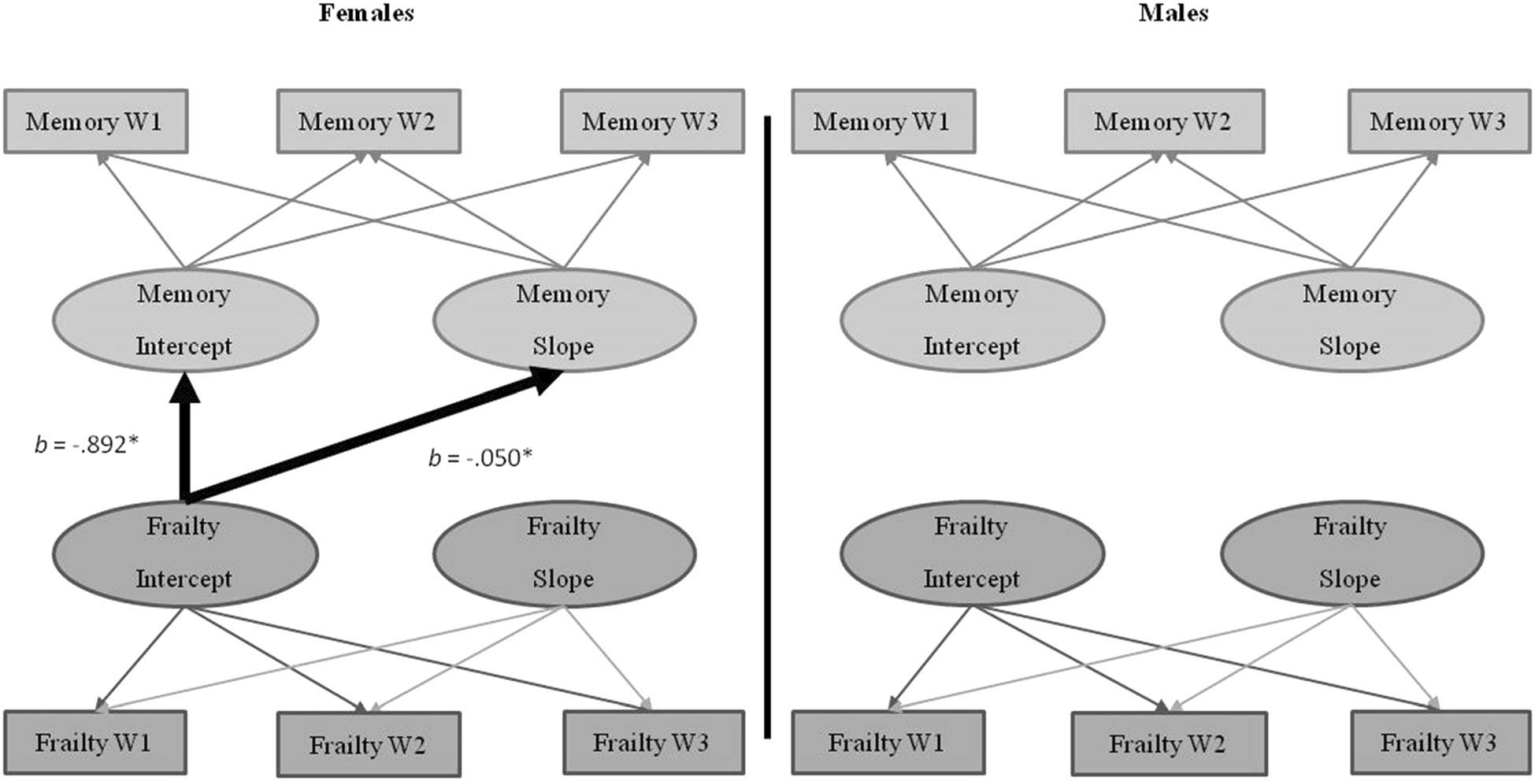
Frailty-memory parallel process model showing moderation by sex. Age in years was the metric of change. The age variable was centered at 75 years. *p < .001

Sex moderated the frailty-memory relationship (*D* = 102.18, Δ*df* = 15, *p* < .001). This moderation occurred for females only. For females, frailty level predicted memory performance (*b* = − .892, *p* = .014) and change in memory (*b* = − 0.050, *p* = 0.013; see Fig. 3). Specifically, for females, higher (worse) frailty was associated with lower memory performance and steeper memory decline than was lower (better) frailty. This effect was not seen for males, as frailty did not predict the level or change in memory.

#### Sex moderation of the frailty-speed relationship

**Fig. 4:**
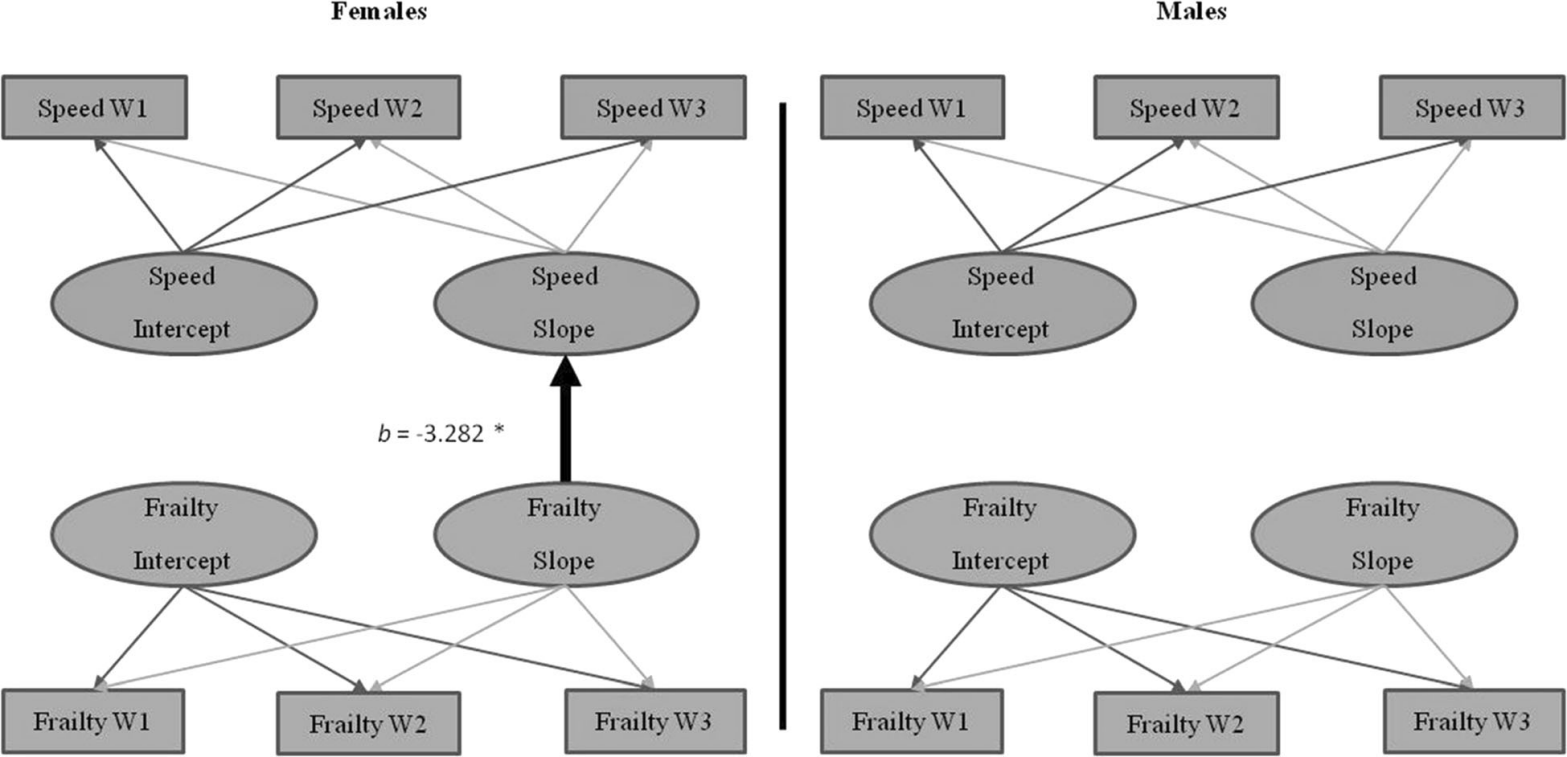
Frailty-speed parallel process model showing moderation by sex. Age in years was the metric of change. The age variable was centered at 75 years. *p < .05

Sex moderated the frailty-speed relationship (*D* = 60.82, Δ*df* = 15, *p* < .001). This moderation occurred for females only; frailty change predicted the change in speed (*b* = − 3.282, *p* = 0.003; see Fig. 4). Specifically, for females, worsening frailty was associated with steeper speed decline than was lower (better) frailty. This effect was not seen for males, as frailty did not predict level or change in speed.

#### Sex moderation of the frailty-EF relationship

**Fig. 5:**
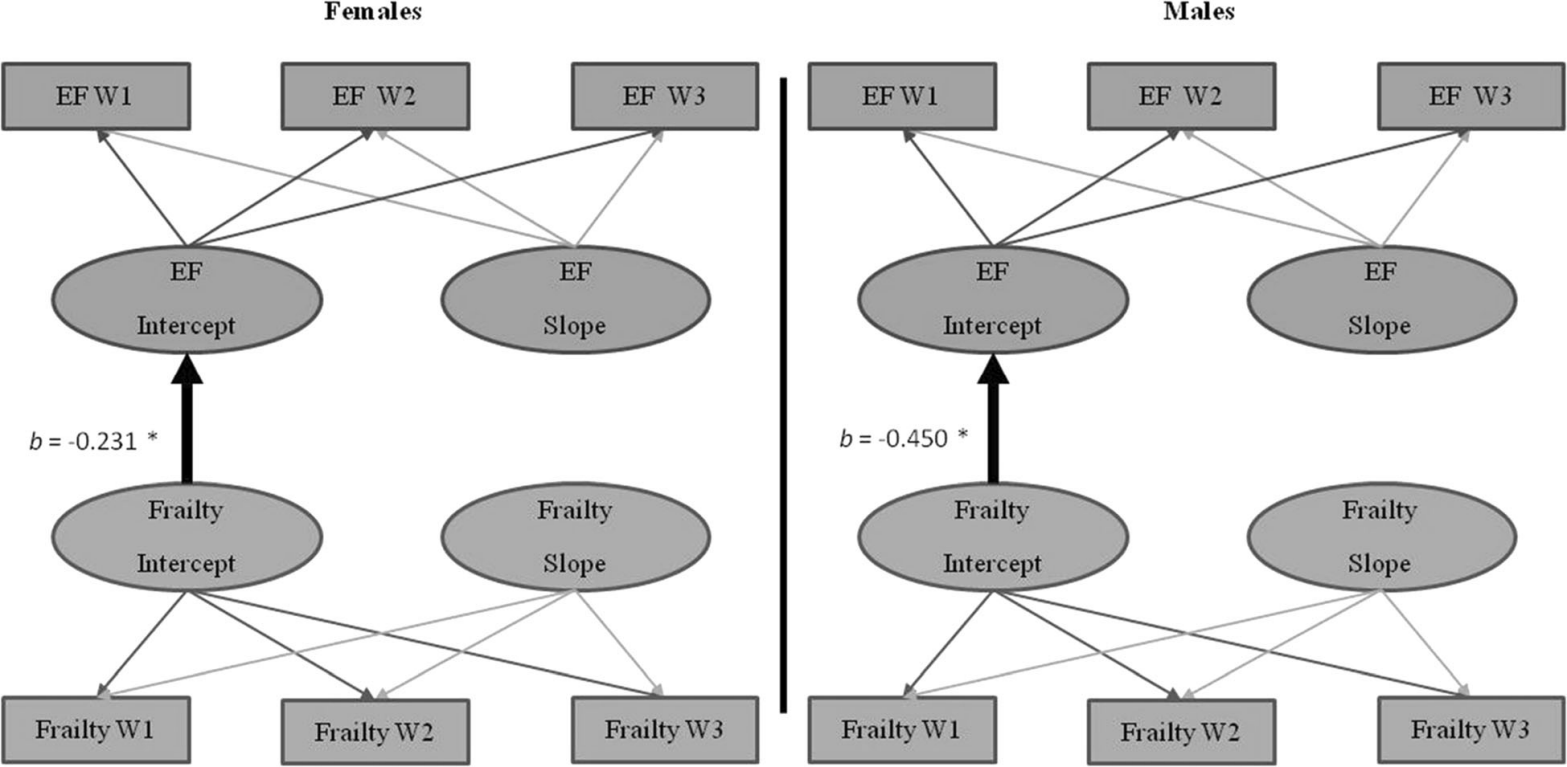
Frailty-EF parallel process model showing moderation by sex. Age in years was the metric of change. The age variable was centered at 75 years. *p < .05

Sex moderated the frailty-EF relationship (*D* = 62.32, Δ*df* = 13, *p* < .001). Frailty level predicted EF performance for both males (*b* = − .450, *p* = 0.029) and females (*b* = − .231, *p* = .048; see Fig. 5). Specifically, higher (worse) frailty was associated with steeper speed decline than was lower (better) frailty for both males and females. As this effect occurred in both sexes, we examined this moderation further. A model with constrained intercept parameters across males and females was a significantly worse fit than the unconstrained model (*D* = 25.7, Δ*df* = 5, *p* < .001). This indicates the effect of frailty on EF was stronger for males than females. Specifically, males with high levels of frailty had lower EF performance than females with the same levels of frailty.

### RG3: Moderation of the frailty-cognition relationships by *APOE*

**Fig. 6:**
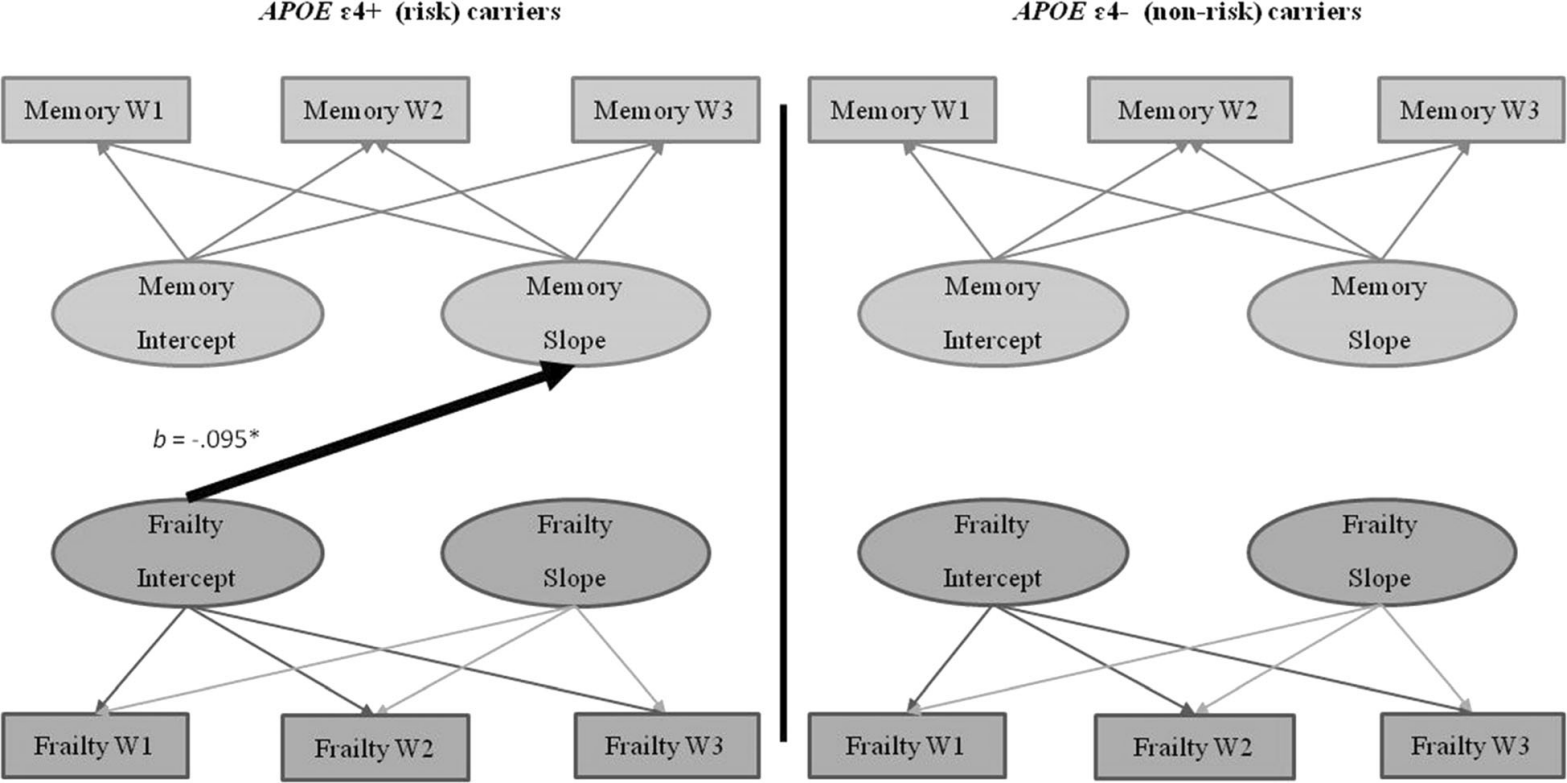
Frailty-memory parallel process model showing moderation by *APOE* status. Age in years was the metric of change. The age variable was centered at 75 years. *p < .05

We conducted six sets of moderation analyses to examine whether *APOE* differentially moderated the previously observed frailty-memory, frailty-speed, and frailty-EF relationships. The results indicated that *APOE* moderated the frailty-memory relationship (*D* = 52.62, Δ*df* = 1, *p* < 0.001). This moderation occurred for the *APOE* risk carriers only. Overall, frailty level predicted the change in memory (*b* = − 0.095, *p* = 0.048; see Fig. 6). Specifically, for *APOE* ɛ4+ (risk) carriers, higher (worse) frailty was associated with steeper memory decline than was lower (better) frailty. *APOE* did not moderate the frailty-speed or frailty-EF relationships.

## Discussion

The overall purpose of this research was to examine the influence of level and change in frailty on three domains of cognition, as moderated by two non-modifiable factors associated with AD. Overall, examining age-related cognitive decline through the lens of the cumulative deficit model indicated that the level of—and worsening—frailty is a major risk factor for both lower cognitive performance and steeper decline. Notably, our results also indicated that the risk factors for AD (i.e., sex and *APOE*) exerted differential effects on the frailty-cognition relationships.

### RG1: Independent effect of frailty on, separately, memory, speed, and EF

We first examined the independent effects of frailty on three domains of cognition, memory, speed, and EF. The results indicated that worse frailty was associated with steeper memory decline. For speed and EF, the results were similar, worse frailty was associated with lower performance, and worsening frailty was associated with steeper decline.

Although expanding, few studies to date have examined the longitudinal relationship between specific cognitive domains and the frailty index. Notably, our results are among the first that examine the relationships between frailty level, change in frailty, and cognitive performance and change. One recent study, using the frailty phenotype, examined baseline frailty as a predictor of performance and change across multiple cognitive domains [48]. Their results indicated that frailty was associated with poorer speed performance, but not speed decline over time; they also found no relationship between baseline frailty and memory performance or change [48]. Our results (using the frailty index) differ in two main ways. First, our results indicated that higher frailty was associated with steeper memory decline, a result not seen by Bunce and colleagues [48]. It is possible that the use of an accumulation of deficit model could delineate the predictive effects on memory not seen using the phenotypic model. Future research could examine and compare the frailty phenotype and frailty index as predictive of longitudinal memory outcomes. Second, our results examined the cognitive influence of frailty level as well as the change in frailty. Specifically, our results indicated a higher frailty level was associated with worse EF and speed performance, a result consistently supported in the literature [13, 48–50]. Notably, however, our results also indicated that an increase in frailty over time was associated with faster EF and speed decline, while a higher frailty level was not. Taken together, these results may indicate that in order to ascertain the influence of frailty on speed or EF change trajectories, it is necessary to examine frailty and cognition as simultaneous change processes.

Neuropathological effects of physical frailty may affect white matter in the posterior and anterior brain regions (associated with EF and speed, respectively) more so than the central white matter regions (associated with memory) [51], a possible explanation for the similar relationships seen between EF and speed. In fact, EF and speed deficits are both found in cognitive impairment associated with dysfunction of the frontal-subcortical circuitry [52] which provides a unifying framework for understanding the functional and cognitive changes associated with neurodegenerative disorders [53]. Notably, multiple age-related complex processes contribute to the development of frailty. Therefore, it is very likely there is a pathophysiologic mechanistic overlap with some of the age-related processes that contribute to the cognitive decline and impairment over the course of the life-span [11]. Indeed, chronic inflammation has been linked to cognitive decline, AD, and frailty [54–56]. Inflammatory receptors located in the hippocampus and prefrontal cortex (associated with memory and EF, respectively) may be adversely affected by the state of chronic inflammation in frailty, affecting EF and memory performance [54, 57], a possible explanation for the results seen for memory and EF. Recent analyses suggest that the deficits that accumulate in a frailty index play an important role not just in dementia risk [2] but also in moderating the relationship between Alzheimer’s neuropathology and the clinical expression of dementia [58]. Those analyses, controlled for sex and *APOE* ɛ4 status, are consistent with what we have observed here.

### RG2: Moderation of the frailty-cognition relationships by sex

The second research goal was to examine sex moderation of frailty-cognition relationships. The results indicated that frailty predicted worse cognitive performance or change across all three cognitive domains for females, but only predicted EF performance level for males. This indicates that females may experience a wider cognitive deficit from higher levels of frailty than males.

Females have a higher risk for AD than males. Additionally, females have been found to have higher levels of frailty than men, but lower levels of mortality [59]. This may be because men may have a lower threshold for deficit accumulation than females; at any level of frailty, men may have changed more from their baseline status [21]. In fact, descriptive analyses indicated that overall, women in our sample had higher frailty levels; however, men at the same frailty level had steeper frailty change trajectories than women, supporting the male-female health-survival paradox, a phenomenon in which females experience higher rates of disability and poor health but longer lives than males [21]. While some recent research has examined the effect of frailty across different domains of cognition, our study is one of the first to specifically examine sex differences within these frailty-cognition associations.

Notably, men in this study did not experience a cognitive cost of frailty on memory or speed performance or change. However, our results indicated the effect of frailty on EF performance was stronger for males than females. A recent study of sex differences in cognition by McCarrey and colleagues [22] indicated that in a cognitively normal sample of older adults, males and females experienced the same rates of decline in EF. Therefore, frailty may be a discriminating factor of cognitive differences between sexes; there may be a higher cognitive cost of frailty for males that result in a more profound EF deficit, despite more widespread cognitive deficits for females. In fact, a recent study by Gallucci and colleagues [60] examined the association between frailty and cerebral atrophy. Their results indicated an increase in frailty was associated with an increase in cortical atrophy in the frontal and temporal lobes, an effect which was more evident in males, despite a similar level in frailty between the two sexes [60]. Taken together, the effect of frailty may have a higher impact on EF performance for males due to the higher level of frontal lobe atrophy that occurs with the accumulation of deficits.

The pathophysiologic underpinnings of frailty may differ between males and females [21, 61]. Among them, inflammatory markers, hormones, and genetic influences have all been found to exhibit both differential and systemic effects on frailty [54, 61–64]. Future research should examine sex differences in physiological biomarkers of frailty, as well as examine frailty-related sex differences in the brain structure and accumulation of neuropathology that could explain the frailty-cognition sex differences seen in this study.

### RG3: Moderation of the frailty-cognition relationships by *APOE*

Research goal three was to examine *APOE* moderation of the frailty-cognition relationships. Our results indicated that *APOE* only moderated the association between frailty and memory. Specifically, for *APOE* risk carriers, frailty predicted significant memory decline, suggesting that genetic risk may increase vulnerability to negative health states, such as cumulative health deficits. *APOE* ɛ4 is recognized as a “frailty allele” [65]. However, the literature examining the relationship between *APOE* and frailty is sparse. One study conducted by Rockwood and colleagues [18] found no relationship between frailty and *APOE* status. Notably, the effect of *APOE* has been found to occur in interaction with health and lifestyle factors [35]. Therefore, more information may be offered when examining *APOE* interactively, rather than as an independent influence.

*APOE* may moderate the relationship between frailty and memory by promoting more widespread neuropathology, particularly in the deeper, medial regions associated with memory before the onset of neurodegenerative disease [66]. Buchman and colleagues [67] found that the accumulation of brain pathology may contribute to frailty progression in older adults. Additionally, Bailey and colleagues [68] found that *APOE* ɛ4 carriers had smaller medial temporal lobe volumes and that the volume mediated the relationship between memory performance and *APOE* genotype. *APOE* ɛ4 is also associated with altered levels of C-reactive protein, a systemic marker of inflammation [69] which has been found to be associated with frailty, [70] memory performance and lower medial temporal volume [71], and cognitive decline in a non-demented population [72]. Taken together, frailty biomarkers, *APOE*, and age-related memory decline may share common pathophysiological mechanisms (i.e., brain atrophy, beta-amyloid burden, inflammatory markers) [73].

There are several strengths and limitations to this study. A first limitation is the participants of the VLS may not be a representative of the broadest population of older adults, as they are initially selected to be relatively healthy, free of neurodegenerative disease, and may possess several risk-reducing factors. However, they could reflect a growing proportion of older adults in western countries. Second, only participants from the first and third VLS cohorts contributed three data points to this particular study. A more complete design would have included three data points from all samples. However, this design characteristic did not affect the results, as evidenced by the invariance testing, which showed that the executive function, episodic memory, and neurocognitive speed latent variables were the same across time and could be compared at each data point. Third, we did not determine the directional effects of the frailty-cognition relationship, as we examined frailty as a predictor of cognitive performance and change. Future research could examine the possibility of bidirectional frailty-cognition relationships. Fourth, we were not able to examine moderation with respect to a sex × *APOE* interaction, as these models did not converge. This non-convergence could be due to a low number of *APOE ɛ4*+ males (*n* = 54) which is not sufficient for the complex analyses used to jointly model the performance and change of both frailty and cognition within this study [74]. Fifth, our present analyses do not model trajectory-based subgroups for either frailty or cognition [75]. Such trajectory subgroup analyses could potentially distinguish patterns of improvement, stability, and decline that would be valuable to investigate in future research [76]. Regarding strengths, first, we used contemporary statistical approaches to systematically analyze three complex research goals, examining (a) longitudinal frailty-cognition relationships using three parallel process growth models and (b) the moderating influence of two major risk factors for AD (i.e., sex, *APOE*). Second, we used multiple standard episodic memory, executive function, and neurocognitive speed variables, which contributed to validated, invariant, longitudinal latent variables. This is valuable as the use of latent variables adjusts for the measurement error that affects the reliability of measurement when using a single measure [47]. Third, we used an accelerated longitudinal design with age as the metric of change, allowing age to be incorporated directly into the analyses. Fourth, we used a substantial and well-characterized longitudinal sample (wave 1, *n* = 632) tested at 3 waves across a band of 40 years of aging. Fifth, we developed a frailty index using 50 non-cognitive and non-genetic variables that previously demonstrated effectiveness in frailty indices.

## Conclusions

In conclusion, we found that physical frailty, as measured by a frailty index, in non-demented older adults affects the performance and change in three age-sensitive cognitive domains. Our results are among some of the first to contribute information about moderation of the cognitive consequences of frailty in non-demented aging [77]. Specifically, two non-modifiable AD biomarkers differentially modified these relationships. Frailty predicted worse cognitive performance or change across all three domains of cognition for females but only for EF for males. An *APOE* moderating effect was evidenced, predicting the rate of memory decline for *APOE* risk carriers only. Our results provide further evidence of the link between frailty and cognitive decline and contribute to the idea that multifactorial mechanisms contribute to cognitive decline. Disentangling the link between frailty and cognition can offer two main benefits, (a) identification of risk factors for cognitive decline and impairment and (b) evidence-based development of new interventions that can target both frailty and cognitive decline [11]. For example, interventions that target a large array of health factors (or overall health status) with a life-course approach [14] may prove to be the best way to prevent or delay cognitive decline and perhaps impairment and dementia.

## Additional file

Additional file 1: Supplementary details, figures, and tables. (DOCX 244 kb)

## Data Availability

The dataset generated and analyzed during the current study is available from the corresponding author on reasonable request.

## Abbreviations

AD: Alzheimer’s disease
AIC: Akaike Information Criteria
APOE: Apolipoprotein E
BIC: Bayesian information criteria
CFI: Comparative fit index
*D*: Deviance statistic (− 2*LL)
DSM-5: Diagnostic and Statistical Manual of Mental Disorders, Fifth Edition
EF: Executive function
LL: Log likelihood
RG: Research goals
RMSEA: Root mean square error of approximation
SEM: Structural equation modeling
SRMR: Standardized root mean square residual
VLS: Victoria Longitudinal Study
