# Supplementary material for: Frailty effects on non-demented cognitive trajectories are moderated by sex and Alzheimer’s genetic risk
Source: Alzheimers Res Ther. 2019 Jun 21;11:55. doi: 10.1186/s13195-019-0509-9 (PMC6587247; doi:10.1186/s13195-019-0509-9)
Supplement: Supplementary file 1 — Supplementary details, figures, and tables. (DOCX 244 kb) [file 13195_2019_509_MOESM1_ESM.docx]

**Supplementary Material for:** Frailty Effects on Non-Demented Cognitive Trajectories are Moderated by Sex and Alzheimer’s Genetic Risk

**Statistical Analyses**

***Longitudinal Measurement Invariance***. Tested using (a) configural invariance, which is used to determine if the same EF measures represent the latent variable at each wave of data collection, (b) metric invariance, which is used to determine that each EF latent variable was measuring the same construct, and (c) scalar invariance, which tests whether there are mean differences at the latent mean level. Model fit was determined using standard indices: (a) χ^2^ for which a good fit would produce a non-significant test (p > .05), indicating the data are not significantly different than the model estimates, (b) comparative fit index (CFI) for which ≥ .95 was judged a good fit and between .90 and .94 was judged an adequate fit, (c) root mean square error of approximation (RMSEA), for which ≤ .05 would be judged good and between .06 and .08 would be judged adequate, and (d) standardized root-mean-square residual (SRMR) for which good fit is judged by a value of ≤ .08 (Kline, 2011; Little, 2013).

***Latent Growth Modeling.*** Growth models were tested in this order: (a) a fixed intercept model, which assumed no inter- or intra-individual variation, (b) a random intercept model, which modeled inter-individual variability in overall level but no intra-individual change, (c) a random intercept fixed slope model, which allowed inter-individual variability in level but assumes all individuals exhibited the same rate of change, and (d) a random intercept, random slope model which allowed inter-individual variability in level and change. When growth modeling uses individually varying times of observation as parameters the traditional SEM model fit indices (i.e., chi-square, RMSEA, CFI, etc) are not available. Therefore, the log-likelihood (LL) and indices of relative fit, the Akaike (AIC) and Bayesian information criteria (BIC), are provided. The LL is the measure of the magnitude of the log-likelihood function for the particular combination of parameter estimates and observed data (Singer & Willett, 2003). It contains all parameters (sample data and the unknown parameters); smaller absolute values mean a better model fit (Singer & Willett, 2003). To compare the nested growth models, the Deviance statistic was used (*D*). The deviance statistic is -2 times the sample LL and measures the discrepancy between the current model and the full model. The difference (Δ*D)* between the a full model and the reduced model has a χ^2^ distribution with degrees of freedom which is the number of constraints imposed (Singer & Willett, 2003). The Δ*D* value was compared to a χ^2^ critical value with the appropriate degrees of freedom; a significant deviance statistic indicates a better model fit than the previous model. Additionally, we evaluated the resulting parameter estimates to ensure that all variances were positive (i.e., no Heywood cases) and the resulting parameter estimates were plausible (Singer & Willett, 2003).

***Parallel Process Models.*** We tested whether frailty predicted memory, speed, or executive function using latent growth curve parallel process models. Latent growth curve parallel process models address the foundational question of whether or not there is an association between growth parameters (Little, 2013). Three parallel process models were tested to see whether (a) level of frailty predicted either level or change in (separately) memory, speed, or EF, and (b) change in frailty predicted change in (separately) memory, speed, or EF. First, we tested the frailty growth model in parallel process with the memory growth model to evaluate whether frailty or change in frailty exerted important effects on level or change in memory. Path analyses were used to determine the effects (a) level of frailty (intercept) regressed on level of memory performance (intercept), (b) level of frailty (intercept) regressed on memory change (slope), and (c) change in frailty (slope) regressed on memory change (slope). These steps were repeated using the frailty growth model in parallel process with the speed growth model, and then with the EF growth model.

**Results**

**Foundational Analyses**

***Confirmatory factor analysis and measurement invariance testing.*** Briefly, a single-factor memory model comprised of four memory manifest indicators fit this sample of participants, and had partial scalar invariance across time (final model fit indices: RMSEA = .07; CFI = .95; SRMR = .08; *Δχ^2^* = 9.88, *Δdf* = 4, *p* = .042). A single-factor speed model comprised of four manifest indicators fit this sample of participants, and had partial scalar invariance (RMSEA = .096; CFI = .94, SRMR = .086; *Δχ^2^* = 94.32, *Δdf* = 2, *p* < .001). A single-factor EF model comprised of four manifest indicators fit the data and had partial scalar invariance (RMSEA = .04; CFI = .97, SRMR = .08; *Δχ^2^* = 44.7, *Δdf* = 4, *p* < .001; see Supplementary Material tables S1-S4 for model fit and model testing comparisons). Partial scalar invariance for all measures indicates mean differences were evident at the factor level and mean level for the majority of the indicators, and we “can proceed with making comparisons of the construct’s key parameters” (Little, 2011, pg. 178).

***Latent growth models for speed, memory, and EF.*** For memory we observed that individuals varied in performance at the centering age (*b* = 17.745, *p* < 0.01), exhibited significant decrease in memory performance (*M* = -0.073, *p* < 0.01), and showed variable patterns of decline (*b* = 0.027, *p* < 0.01; see Supplementary Material Figure S2). Third, for speed we observed that individuals varied in level of speed performance at age 75 (*b* = 67.849, *p* < 0.01), exhibited significant decrease in performance (*M* = -0.100, *p* < 0.01), and showed variable patterns of decline (*b* = 0.139, *p* < 0.01; See Supplementary Material Figure S3). Fourth, for EF, we observed that individuals varied in level of performance at the centering age (*b* = 0.997, *p* < 0.01), exhibited significant decrease in EF performance (*M* = -0.012, *p* = 0.01), and showed variable patterns of decline (*b* = 0.003, *p* < 0.01; See Supplementary Material Figure S4).

Table S1

Growth Model Goodness of Fit Index for Frailty

*Note.* -2LL = -2 Log likelihood; AIC = Akaike information criterion; BIC = Bayesian information criterion; *D* = difference statistic (using -2LL); Δdf = change in degrees of freedom; -2LL = -2 Log likelihood

* Best fitting model

Table S2

Goodness of Fit Indices for **Episodic Memory (EM**) Confirmatory Analysis Models and Measurement Invariance Testing

Note. CFA = Confirmatory Factor Analysis; AIC = Akaike information criterion; BIC = Bayesian information criterion; χ2 = chi-square test of model fit; df = degrees of freedom for model fit; RMSEA = Root Mean Square Error of Approximation; CFI = Comparative Fit Index; SRMR = Standardized Root Mean Square Residual; Δ χ2 = change in chi-square; Δdf = change in degrees of freedom; -2LL = -2 Log likelihood; D = difference statistic (using -2LL)

*Best Fitting Model

a RAVLT free recall and RAVLT recall after interference free to vary

Table S3

Goodness of Fit Indices for **Neurocognitive Speed** Confirmatory Analysis Models and Measurement Invariance Testing

*Note.* CFA = Confirmatory Factor Analysis; AIC = Akaike information criterion; BIC = Bayesian information criterion; χ^2^ = chi-square test of model fit; df = degrees of freedom for model fit; RMSEA = Root Mean Square Error of Approximation; CFI = Comparative Fit Index; TLI = Tucker-Lewis Index; SRMR = Standardized Root Mean Square Residual; Δ χ^2^ = change in chi-square; Δdf = change in degrees of freedom; -2LL = -2 Log likelihood; *D* = difference statistic (using -2LL)

*Best Fitting Model

^a^ Simple Reaction Time, Lexical Decision, and Sentence Verification free to vary

Table S4

Goodness of Fit Indices for **Executive Function (EF**) Confirmatory Analysis Models and Measurement Invariance Testing

*Note*. CFA = Confirmatory Factor Analysis; AIC = Akaike information criterion; BIC = Bayesian information criterion; χ2 = chi-square test of model fit; df = degrees of freedom for model fit; RMSEA = Root Mean Square Error of Approximation; CFI = Comparative Fit Index; TLI = Tucker-Lewis Index; SRMR = Standardized Root Mean Square Residual; Δ χ2 = change in chi-square; Δdf = change in degrees of freedom; -2LL = -2 Log likelihood; D = difference statistic (using -2LL)

*Best Fitting Model

^a^ Brixton and Color Trails free to vary


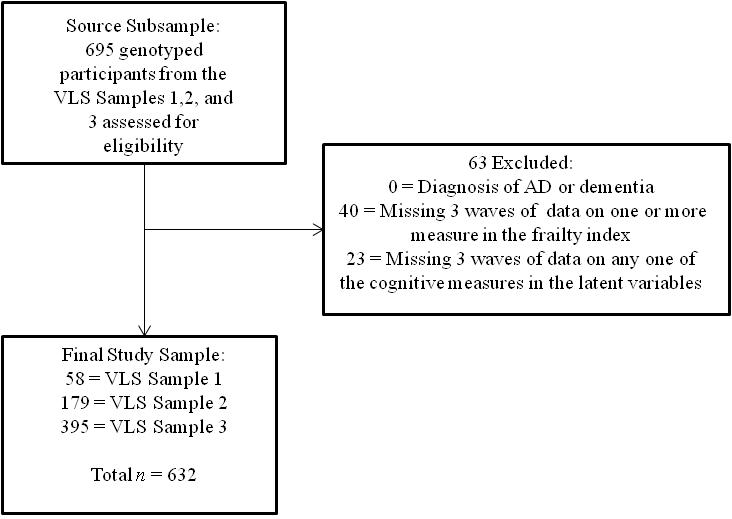


*Figure S1.* Study flowchart of study participants. The final sample consisted of 632 participants.


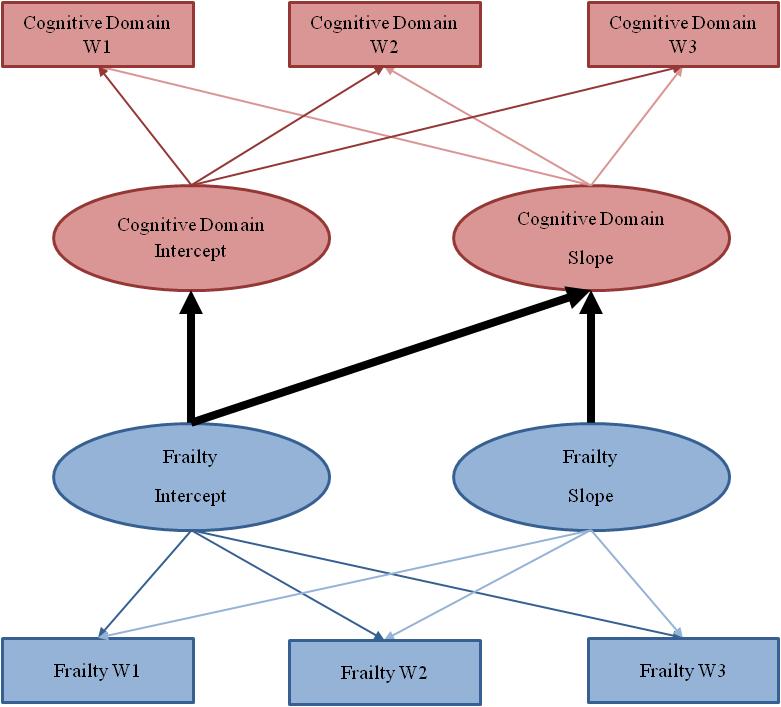


*Figure S2.* Illustration of the Frailty-Cognition parallel process model, in which level of frailty is regressed onto level and slope of cognition, and slope of frailty is regressed onto the slope of cognition.


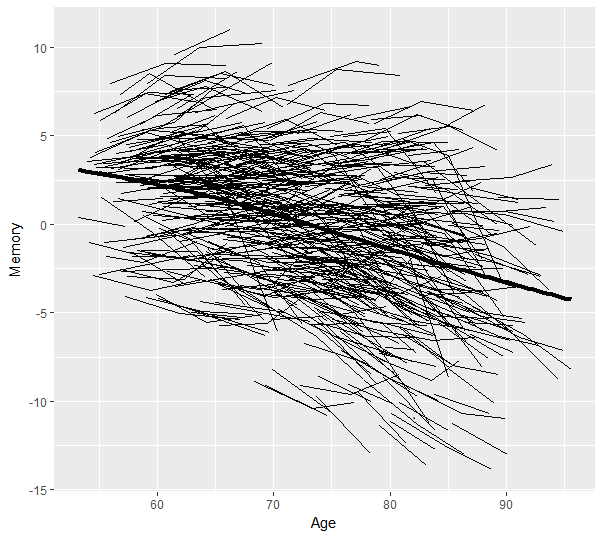


*Figure S3.* Individual memory trajectories across a 40-year band of aging with a group mean trajectory line (final growth model random intercept, random slope; *D* = 342.02, Δdf = 2, *p* <.001).

*
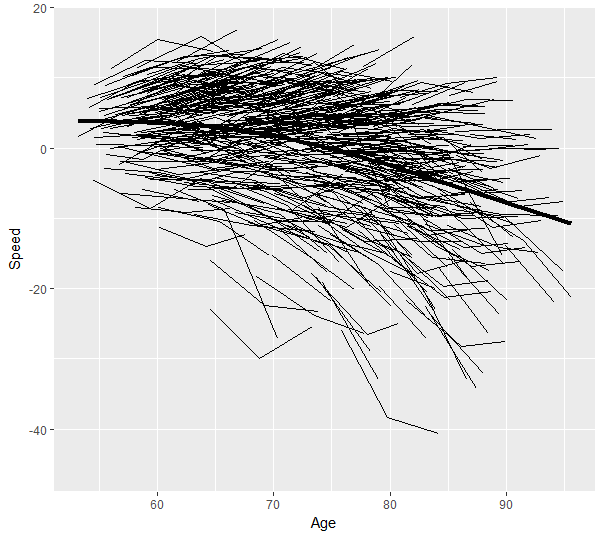
*

*Figure S4.* Individual speed trajectories across a 40-year band of aging with a group mean trajectory line (final growth model random intercept, random slope; *D* = 444.58, Δdf = 2, *p* <.001).

*
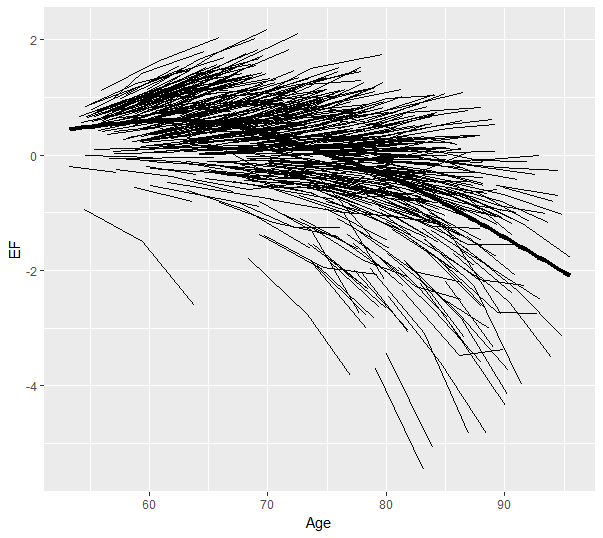
*

*Figure S5.* Individual Executive Function (EF) trajectories across a 40-year band of aging with a group mean trajectory line (final growth model random intercept, random slope; *D* = 838.0, Δdf = 2, *p* <.001).

References

Kline, R. B. (2011). *Principles and practice of structural equation modeling* (Vol. 3rd). New York, NY: Guilford Press.

Little, T. D. (2013). *Longitudinal structural equation modeling*. New York, NY: Guilford Press.

Singer, J. D., & Willett, J. B. (2003). *Applied longitudinal data analysis: Modeling change and event occurrence*. New York, NY: Oxford University Press; US.
